# Lesions causing post-stroke spasticity localize to a common brain network

**DOI:** 10.3389/fnagi.2022.1011812

**Published:** 2022-10-25

**Authors:** Yin Qin, Shuting Qiu, Xiaoying Liu, Shangwen Xu, Xiaoyang Wang, Xiaoping Guo, Yuting Tang, Hui Li

**Affiliations:** ^1^Department of Rehabilitation Medicine, The 900th Hospital of Joint Logistic Support Force, People’s Liberation Army (PLA), Fuzhou, China; ^2^Department of Rehabilitation Medicine, Fuzong Clinical Medical College of Fujian Medical University, Fuzhou, China; ^3^College of Rehabilitation Medicine, Fujian University of Traditional Chinese Medicine, Fuzhou, China; ^4^Department of Radiology, The 900th Hospital of Joint Logistic Support Force, People’s Liberation Army (PLA), Fuzhou, China

**Keywords:** post-stroke spasticity, lesion network mapping, functional connectivity, lesions, resting-state networks

## Abstract

**Objective:**

The efficacy of clinical interventions for post-stroke spasticity (PSS) has been consistently unsatisfactory, probably because lesions causing PSS may occur at different locations in the brain, leaving the neuroanatomical substrates of spasticity unclear. Here, we investigated whether heterogeneous lesions causing PSS were localized to a common brain network and then identified the key nodes in this network.

**Methods:**

We used 32 cases of PSS and the Human Connectome dataset (*n* = 1,000), using a lesion network mapping method to identify the brain regions that were associated with each lesion in patients with PSS. Functional connectivity maps of all lesions were overlaid to identify common connectivity. Furthermore, a split-half replication method was used to evaluate reproducibility. Then, the lesion network mapping results were compared with those of patients with post-stroke non-spastic motor dysfunction (*n* = 29) to assess the specificity. Next, both sensitive and specific regions associated with PSS were identified using conjunction analyses, and the correlation between these regions and PSS was further explored by correlation analysis.

**Results:**

The lesions in all patients with PSS were located in different cortical and subcortical locations. However, at least 93% of these lesions (29/32) had functional connectivity with the bilateral putamen and globus pallidus. These connections were highly repeatable and specific, as compared to those in non-spastic patients. In addition, the functional connectivity between lesions and bilateral putamen and globus pallidus in patients with PSS was positively correlated with the degree of spasticity.

**Conclusion:**

We identified that lesions causing PSS were localized to a common functional connectivity network defined by connectivity to the bilateral putamen and globus pallidus. This network may best cover the locations of lesions causing PSS. The putamen and globus pallidus may be potential key regions in PSS. Our findings complement previous neuroimaging studies on PSS, contributing to identifying patients with stroke at high risk for spasticity at an early stage, and may point to PSS-specific brain stimulation targets.

## Introduction

Post-stroke spasticity (PSS), one of the most common complications in patients with stroke, persists in approximately 17–42.6% of patients by 3 months after the onset of stroke (Urban et al., 2010; Zorowitz et al., 2013). It is characterized by a velocity-dependent increase in the stretch reflex, with hyperactive tendon reflexes (Lance, 1980). Spasticity gradually worsens over time, resulting in decreased joint mobility, joint contracture, and deformity, which seriously hinders the recovery of limb function and reduces the quality of life (Sommerfeld et al., 2004; Pundik et al., 2019). Therefore, early intervention may help reduce the severity of spasticity and its long-term complications (Wissel et al., 2015). At present, various clinical treatment methods have focused on the management and treatment of PSS, but the effects are not satisfactory (Chih-Lin and Hu, 2018; Sun et al., 2019; Hu et al., 2021). The biggest challenge is that the mechanism of PSS occurrence is not clearly understood, and patients at high risk of spasticity cannot be identified at an early stage.

Previous studies have attempted to clarify the relationship between the location of stroke-associated brain lesions and the occurrence of spasticity, suggesting that PSS may be directly caused by the lesion. Using voxel-based lesion-symptom mapping (VLSM), some researchers found that the basal nucleus, cerebellum, insular lobe, thalamus, and white matter bundle (internal capsule, radiation crown, outer capsule, and superior longitudinal bundle) are closely related to PSS (Cheung et al., 2016; Lee et al., 2019), suggesting that PSS lesions are located at different locations in the brain. Further research has indicated that PSS is a type of network dysfunction disease; that is, the occurrence of spasticity is not only related to the damage of a specific brain structure, but may be related to the reorganization of the distant cortex and abnormalities of the whole brain network after brain injury (Norris et al., 2020; Brüggemann, 2021). Thus, the relationship between lesion locations and spasticity may also arise from the indirect effects of lesions on remote but linked brain areas, a concept known as diaschisis (Carrera and Tononi, 2014). This may reflect the impact of the lesion on broader polysynaptic connections and functional relationships between brain regions. However, the value of traditional lesion mapping methods, such as VLSM, is limited in terms of mapping symptoms resulting from network dysfunction given its nature. Since this method only focuses on the location of the lesion, it does not consider the interconnected areas far from the lesion and may miss some key and unique information. The location of lesions showed significant heterogeneity in different patients with PSS, making it difficult to locate PSS to a common brain region (Picelli et al., 2014; Barlow, 2016). Consequently, the specific neuroanatomical substrates of PSS remain unknown.

Recently, with the introduction of a novel method termed “lesion network mapping,” it has been feasible to map neuropsychic symptoms to brain networks depending on the location of the lesion causing the symptoms and the human connectivity map (Boes et al., 2015). This method is based on the concept of diaschisis, which can determine brain regions or networks specific to symptoms by comparing the functional connectivity characteristics of lesions causing symptoms. The resulting lesion network map may include all the locations of the lesions causing the symptom, which is helpful in prioritizing prevention or early treatment of patients at high risk. Some studies have shown that the results of lesion network mapping are promising as therapeutic targets for invasive or non-invasive brain stimulation (Joutsa et al., 2018b; Joutsa et al., 2019; Ganos et al., 2022). In addition, this method only requires routine clinical magnetic resonance imaging (MRI) scanning to detect the locations of the lesions, which obviates the need for functional brain imaging in patients and is more feasible. It has been proven in many different neuropsychiatric syndromes, including cervical dystonia (Corp et al., 2019), hemichorea-hemiballismus (Laganiere et al., 2016), parkinsonism (Joutsa et al., 2018a), and depression (Padmanabhan et al., 2019). However, no studies have reported the lesion network associated with PSS.

Therefore, we used resting-state functional MRI (fMRI) data of healthy subjects from a large normative dataset and then used lesions from patients with PSS as seed regions for functional connectivity analysis. We identified brain regions and networks specific to PSS using lesion network mapping methods. We hypothesized that lesions causing PSS would be localized to a common brain network that may contain all lesions that contribute to PSS.

## Materials and methods

### Participants

The participants were patients with hemiplegia after ischemic stroke treated at the 900th Hospital of the Joint Logistics Support Force. Inclusion criteria were: (i) ischemic stroke confirmed by MRI; (ii) first onset and course of disease > 3 months; (iii) age from 45 to 75 years; (iv) with unilateral limb motor dysfunction; (v) stable vital signs and clear consciousness; and (vi) signed informed consent and voluntary participation. Exclusion criteria were: (i) severe stroke with extensive brain injury; (ii) presence of metal implants or pacemakers in the body; (iii) other neurological or musculoskeletal disorders that may affect spasticity assessment; and (iv) current or previous use of anti-spasticity drugs or therapies that may affect spasticity (e.g., botulinum toxin, phenol blocks). Based on the modified Ashworth score (MAS), patients with post-stroke motor dysfunction were divided into the spasticity group (MAS score ≥ 1) and non-spastic group (MAS score = 0). We finally enrolled 32 patients with PSS (mean age: 59.28 ± 10.37 years; male: 17 cases) and 29 patients with post-stroke non-spastic motor dysfunction (mean age: 60.76 ± 9.82 years; male: 14 cases). The demographics of all stroke patients are shown in Table 1. The study was approved by the Medical Ethics Committee of the 900th Hospital (No. 2015011), and all participants provided informed consent.

**TABLE 1:** Demographic characteristics of stroke patients.

Variables	PSS patient (*n* = 32)	Non-spastic patient (*n* = 29)	*T/Z/*χ^2^	*P*
Age, y	59.28 ± 10.37	60.76 ± 9.82	–0.570	0.571
Gender, men/female	17/15	14/15	0.143	0.705
Education, y	6 (3.3, 9.0)	6 (4.5, 8.5)	–0.073	0.942
Course of disease, m	4 (3.1, 5.3)	4 (3.0, 5.0)	–0.830	0.407
Side of lesion, left/right	18/14	16/13	0.007	0.933
Modified Ashworth Scale	1.56 (1, 2)	0	–	–

Continuous data are presented as the (mean ± SD) or median (quartile) according to the normal distribution test.

### Magnetic resonance imaging data acquisition

The MRI data of all stroke patients were acquired using a 3.0-Tesla Tim Trio Siemens scanner with a 12-channel head coil. Whole-brain structural images were scanned using fast gradient-echo sagittal three-dimensional T1-weighted image sequences: repetition time (TR)/echo time (TE) = 1,900/2.52 ms, flip angle (FA) = 90°, slice thickness = 1 mm, no gap, slices = 176 and matrix size = 256 × 256. High-resolution anatomical images were scanned using fast spin-echo T2-weighted images sequences: TR/TE = 3,300/98 ms, FA = 120°, slice thickness = 4 mm, slices = 32, slice gap = 0.8 mm, and matrix size = 256 × 320. Resting-state functional MRI data were acquired using a single-shot echo planar imaging sequence: TR/TE = 2,000/21 ms, slice thickness = 4 mm, slices = 33, slice gap = 0.8 mm and matrix size = 64 × 64.

Resting-state fMRI data of 1,000 healthy subjects (mean age: 21.3 years, 57.3% female) were obtained from the Brain Genomics Superstruct Project^1^ (Buckner et al., 2014; Holmes et al., 2015). A 3.0-Tesla Siemens scanner was used to perform a 6.2-min resting-state fMRI scan (TR/TE = 3,000/30 ms, FA = 85°, 47-layer axial slice, interleaved acquisition, no gap, and volumes = 124).

### Data preprocessing

The resting-state fMRI data of 1,000 healthy subjects and 20 patients in the spasticity group were preprocessed using Statistical Parametric Mapping (SPM12)^2^ in MATLAB software (R2013a, MathWorks) according to the strategy of Fox (Fox et al., 2005). Briefly, it included the following steps. First, the first four time points were excluded, and the remaining volumes were subjected to slice timing and motion correction. Second, the images were aligned to the functional image template of the Montreal Neurological Institute (MNI) space, and smoothed with a 6-mm full-width-at-half-maximum Gaussian kernel. Finally, regression interference variables (motion parameters, average white matter, global signal, cerebrospinal fluid), and bandpass filtering (0.01 − 0.1 Hz) were performed.

### Lesion network mapping

**FIGURE 1:**
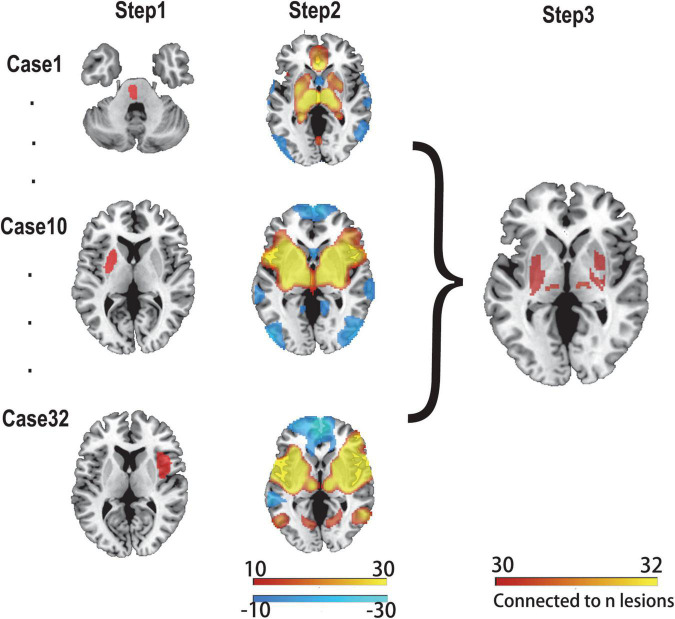
Lesion network mapping method. In step 1, lesions were manually traced to standard brain atlas (MNI 152 template). In step 2, using a resting-state functional connectome dataset (*n* = 1,000), the lesion location was used as a seed point for functional connectivity analysis with the whole brain. The functional connectivity map of each lesion was obtained. In step 3, functional connectivity maps of all lesions were thresholded and binarized, and then overlapped to identify the common brain regions.

We applied a previously proven method for lesion network mapping in three steps to determine whether lesions causing PSS localized to a common brain network (Boes et al., 2015; Figure 1).

First, two experienced radiologists used MRIcron software^3^ to manually delineate the lesions of all patients with PSS on T2 images, and the results were averaged. Each lesion was then standardized to the MNI space.

Second, the 32 lesions causing spasticity were used as seed regions in seed-based resting-state functional connectivity analysis. The fMRI data of 1,000 healthy subjects were used to calculate the time-course correlation between each lesion mask and the whole brain. The correlation graph was transformed into a Z-graph by using Fisher’s R-to-Z transformation. A one-sample *t*-test was used for the random effects analysis to test the group effect. A connectivity t-map was generated for each lesion.

Finally, a threshold of T ≥ ± 7 [equivalent to a whole-brain voxel-based family-wise error (FWE) correction of *p* < 10^–6^] was applied to each of the 32 independent lesion-seeded connectivity network maps to create a binarized map. To ensure that the findings were not influenced by the threshold settings, we used alternative thresholds (T ≥ ± 5 and T ≥ ± 9) for repeated validations (Cohen and Fox, 2020; Sperber and Dadashi, 2020). Next, all binarized graphs were overlapped to identify the common regions connected to the lesion locations associated with PSS.

### Split-half replication

We evaluated the reproducibility of our results by randomly dividing the 32 patients with PSS into two subgroups of 16 patients. The same T-score threshold was used for each subgroup to identify the connected area after repeating the lesion network mapping approach described above.

### Specificity analyses

We compared the lesion networks of patients with PSS and those of patients with post-stroke non-spastic motor dysfunction to assess the specificity of our lesion network mapping findings. We repeated the lesion network mapping method described above for lesions in non-spastic patients. To compare the findings of the lesion network mapping between the PSS and non-spasticity groups, we used two different statistical methods (Ferguson et al., 2019): the Liebermeister test and two-sample *t*-test. The Liebermeister test, which is frequently employed in lesion analysis, categorizes voxels in a binary manner (connected or disconnected). The *t*-test, which is more frequently employed in functional neuroimaging studies, considers connectivity strength. Both tests were included, avoiding the limitation of using either of these two tests alone, and ensuring that the results were robust to different statistical analysis methods.

First, a two-sample *t*-test was implemented using SPM12 software^4^ to compare the connectivity strength of each voxel between the lesions causing PSS and control lesions. Then, the Liebermeister test was performed using MRIcron software to compare the binarized lesion connectivity maps, only voxels involved in at least 10% of all patients with PSS were considered. Significant results were defined as FWE-corrected *p* < 0.05.

### Conjunction analyses and defining the lesion network of post-stroke spasticity

The positive results of the specificity analyses showed that there was a statistical difference between the PSS and non-spastic groups. However, it was unclear how many lesions in the PSS group displayed this result (sensitivity). We then conducted conjunction analyses to pinpoint both sensitive and specific regions associated with PSS. The results determined by the conjunction analysis were then used as regions of interest (ROIs) for the functional connectivity analysis of the whole brain. The functional connectivity map was thresholded according to T ≥ ± 7 (FWE-corrected *p* < 10^–6^) and all voxels connected to the ROIs were identified. The final functional connectivity map obtained was called the lesion network map of PSS.

### Correlation analysis

Pearson correlation analysis was used to determine the correlation of bilateral putamen and globus pallidus with the degree of spasticity (MAS score) to further validate the association of key brain regions with PSS derived from our conjunction analyses. We used resting-state fMRI data from 20 patients with PSS, and the lesions were used as seed points to make functional connectivity with the whole brain. Then, the mean time courses of the right putamen and globus pallidus ROI, left putamen and globus pallidus ROI, and bilateral putamen and globus pallidus were extracted for correlation analysis with patients’ MAS scores. MAS scores 0, 1, 1.5, 2, 3, and 4 indicate the degree of spasticity, with higher scores indicating more severe spasticity. *P* < 0.05 was considered a statistically significant difference.

## Results

### Demographics and lesion locations

**FIGURE 2:**
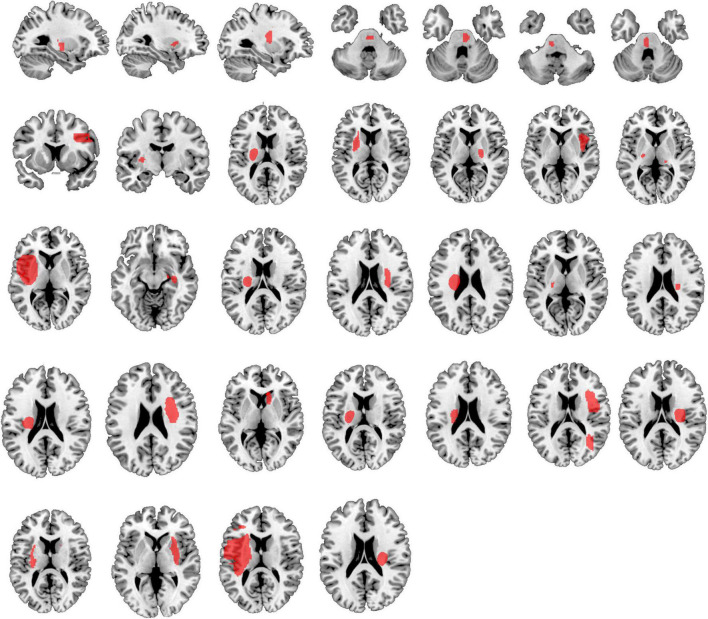
Lesion locations causing PSS. The lesions of 32 patients with PSS were manually located on standard brain atlas. All lesions were tracked according to their laterality.

Table 1 provides detailed demographics of PSS and non-spastic patients. There were no statistical differences between the PSS and the non-spastic group in the field of sex, age, years of education, course of disease, or side of lesion (*p* > 0.05). We identified 32 lesions causing first onset PSS. The lesions occurred at different locations in the brain, including the insula, putamen, thalamus, frontal lobe, pons, parietal lobe, caudate nucleus, globus pallidus, occipital lobe, internal capsule, and paraventricular white matter. Some of patients had multiple lesions. Representative sections of each lesion are shown in Figure 2.

### Lesion network mapping of post-stroke spasticity

**FIGURE 3:**
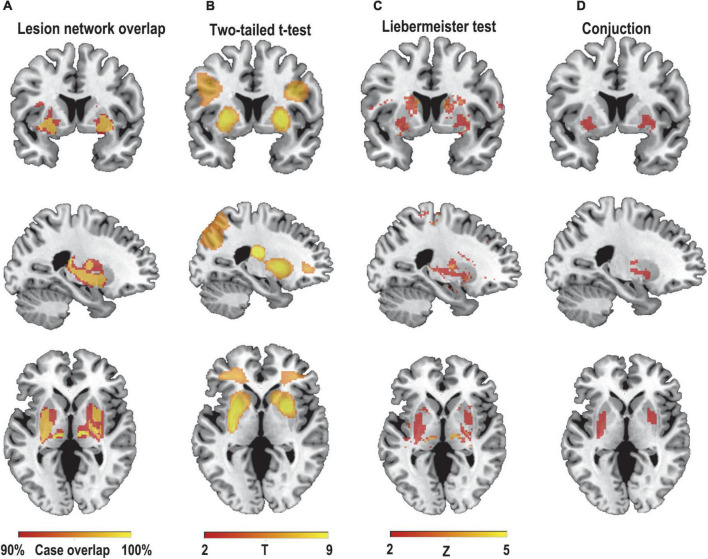
Lesion network mapping of PSS. **(A)** > 93% (30/32) of lesion locations causing PSS were functionally positively connected to the bilateral putamen, pallidum, and thalamus. Using two different statistical methods, two-sample *t*-test **(B)** and Liebermeister test **(C)**, lesion network maps of patients with PSS were compared with those of patients with post-stroke non-spastic motor dysfunction to identify regions specific to PSS (FWE-corrected *p*<0.05). **(D)** The conjunction analyses determined bilateral putamen and pallidum as regions associated with PSS that were both sensitive and specific.

**FIGURE 4:**
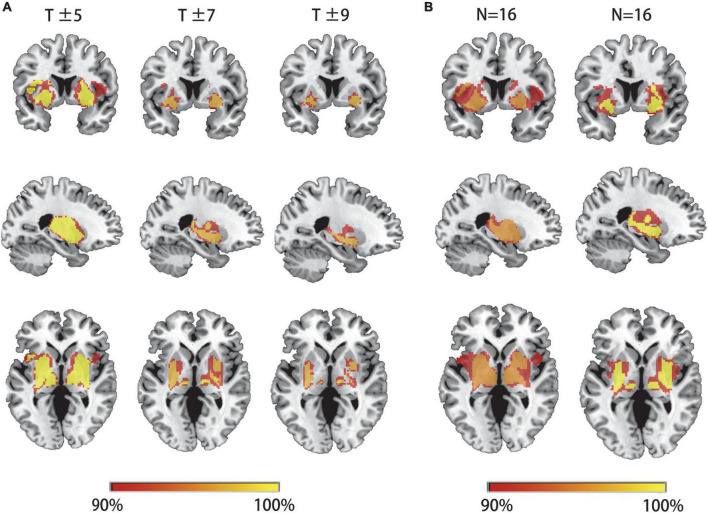
Lesion network mapping results are independent of thresholds and are highly repeatability. **(A)** The overlapping regions of peak lesion networks in the bilateral putamen, pallidum and thalamus were similar in different network thresholds. **(B)** Patients with PSS were randomly divided into two subgroups for comparison of lesion network mapping results. The lesion network overlap regions in both subgroups were found to be highly repeatability and consistent with our results above.

We first hypothesized that lesions causing PSS would be mapped to a single common brain network. To test this hypothesis, a novel approach known as lesion network mapping was applied to identify brain areas with functional connectivity to each patient’s lesion location. Despite the heterogeneity of lesion locations, the lesions causing PSS belong to a common functional connectivity brain network. Our study found that more than 93% of the lesion locations causing PSS (30/32) were functionally positively associated with the bilateral putamen, globus pallidus and thalamus (Figure 3A). The results remained unchanged when different thresholds were used (T ≥ ± 5 and T ≥ ± 9) (Figure 4A). To evaluate the internal reliability of our findings, patients with PSS were randomly divided into two subgroups to compare the lesion network mapping results. We found that more than 93% of lesion locations causing PSS in both subgroups were functionally connected to the bilateral putamen, globus pallidus and thalamus, consistent with our results above and with high repeatability (Figure 4B). Split-half replication proved the consensus positioning of the network overlap regions in individual subgroups.

### Network localization specific to post-stroke spasticity

To identify if these connections were specific to lesions causing PSS, lesion network maps of patients with PSS were compared with those of patients with post-stroke non-spastic motor dysfunction using two statistical methods. Compared to controls, the bilateral putamen and globus pallidus showed consistent connectivity with lesions causing PSS and were independent of statistical methods (FWE-corrected *p* < 0.05) (Figures 3B,C).

### Lesions causing post-stroke spasticity belong to a common network

**FIGURE 5:**
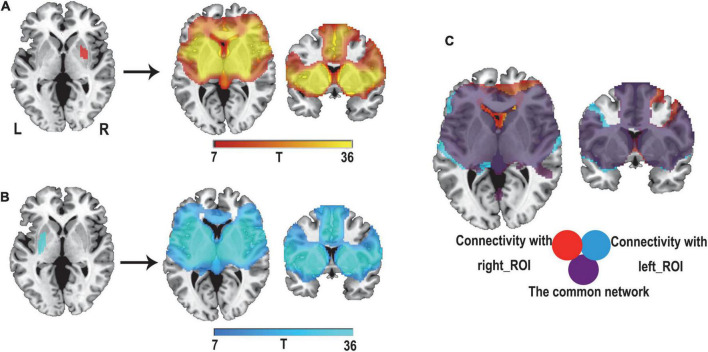
Defining the lesion network of PSS. Positive connectivity with our right putamen and globus pallidus ROI (red) **(A)** and left putamen and globus pallidus ROI (blue) **(B)** were identified, respectively. **(C)** The overlap of these two networks (purple) defines a distributed brain network, which is the lesion network of PSS.

**FIGURE 6:**
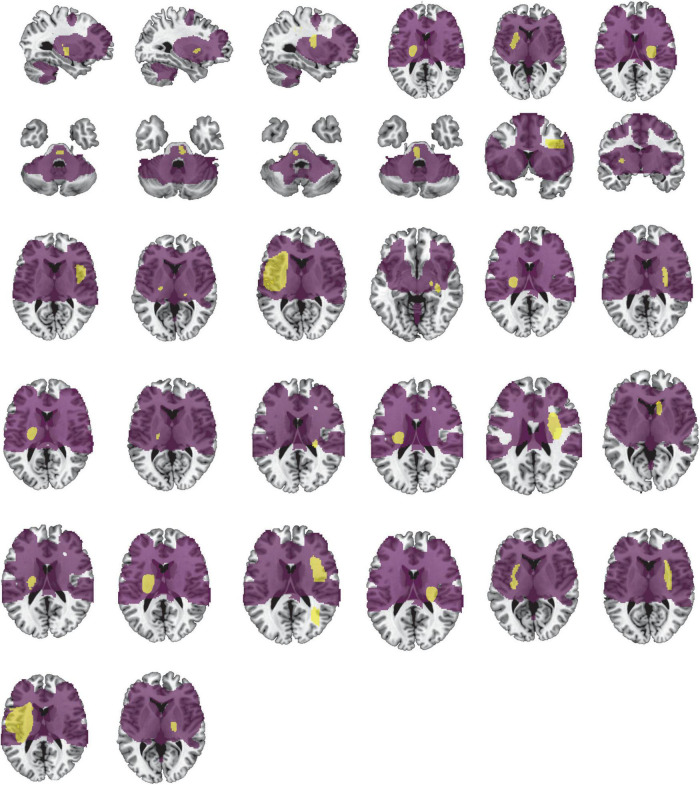
Lesion network involves the location of lesions causing PSS. Our lesion network of PSS involves the location of lesions in 31/32 patients identified in the PSS group. Lesions of PSS (yellow) are the same as in Figure 2. The lesion network of PSS is shown in purple.

The conjunction analyses determined two ROIs that were both sensitive (connected to > 93% of lesions causing PSS) and specific (voxel presence in both specificity tests) to cause PSS: the right putamen and globus pallidus ROI, and the left putamen and globus pallidus ROI (Figure 3D). According to previous definitions, positive connectivity to these ROIs was defined as a distributed network that encompassed all lesion locations contributing to PSS. For visualization, we performed functional connectivity analysis with the whole brain using these two ROIs as seed points to compute all voxels that were positively connected to the bilateral putamen and globus pallidus. We referred to the final functional connectivity map as the lesion network map of the PSS (Figure 5). For illustrative purposes, the lesion locations were superimposed on the lesion network map. As expected, lesions causing PSS belonged to a common functional connectivity network. The network covered the location of lesions in 31/32 patients identified in our PSS group (Figure 6).

### Correlation analysis between post-stroke spasticity and the putamen and globus pallidus

**FIGURE 7:**
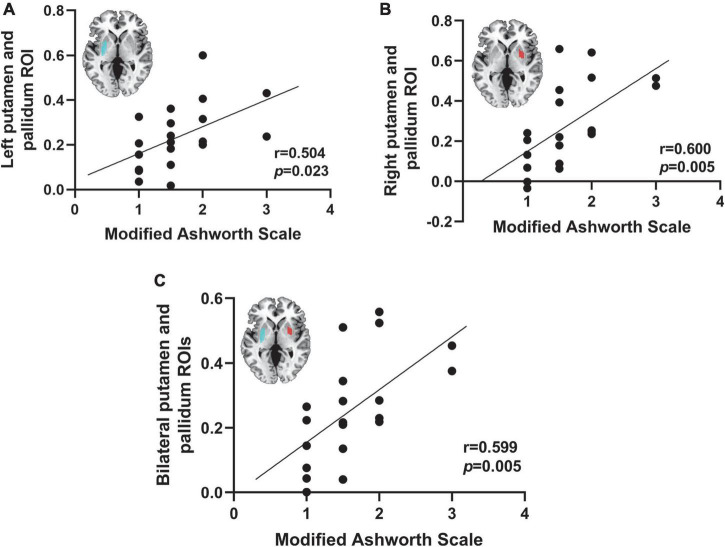
Correlation between spasticity and the putamen and globus pallidus. The functional connectivity of the right putamen and globus pallidus ROI **(A)**, the left putamen and globus pallidus ROI **(B)**, and the bilateral putamen and globus pallidus **(C)** to lesions causing PSS was positively correlated with the MAS score.

We correlated the functional connectivity of lesions to the putamen and globus pallidus was correlated with MAS scores in 20 patients with PSS. The functional connectivity of the putamen and globus pallidus to lesions causing PSS was found to be positively correlated with the MAS score (left ROI: *r* = 0.504, *p* = 0.023; right ROI: *r* = 0.600, *p* = 0.005; bilateral: *r* = 0.599, *p* = 0.005) (Figure 7).

## Discussion

In this study, we employed a lesion network mapping method to study brain regions and networks related to PSS. This study provided new insights into the neural basis of PSS. First, the lesions causing PSS were found to be located in different brain regions, but belonged to a common functional connectivity network. Second, the network was defined by positive connectivity to the bilateral putamen and globus pallidus. These connections were specific to PSS lesions as compared to non-spasticity lesions. This network may best cover the location of lesions that contribute to PSS. Furthermore, the functional connectivity of the putamen and globus pallidus to lesions causing PSS was positively correlated with the degree of spasticity. Our study complements previous neuroimaging studies on PSS and provides insight into the neuroanatomical basis of PSS. These findings may help to identify stroke patients at high risk for spasticity at an early stage and may have potential significance for the identification of therapeutic targets for brain stimulation in PSS.

Owing to adaptive alterations in interconnected brain networks or distant effects of the lesion on linked brain areas, the causal link between lesion locations and symptoms may be indirect (Kim et al., 2021). The traditional lesion mapping method is limited to establishing the location of the lesion and fails to link heterogeneous lesions. Functional neuroimaging has been considered a potential means of studying the wider network effects of brain damage, particularly fMRI-based resting-state functional connectivity (Mirzaei and Adeli, 2016). In this study, based on the lesion network mapping technique, we used the resting-state fMRI of 1,000 healthy individuals to calculate the brain regions connected to the lesions causing PSS. We determined several brain areas, such as the putamen, globus pallidus, and thalamus, that had already been pointed by previous neuroimaging studies. These findings provide strong consistent evidence, indicating that these brain regions are closely related to PSS. Lesion network mapping allows identification of a broader network that is functionally connected to lesion locations, providing information on the possible connectivity patterns of the lesion location before the onset of symptoms and linking anatomically distinct lesions to cortical areas associated with the same symptoms. Expanding the positioning of symptoms from focal lesion locations to focal lesion networks will be significantly complement lesion mapping methodology. This study goes beyond previous work by examining the network of lesions causing PSS, and it did not require *a priori* assumptions about the exact location of the network.

Previous studies have considered PSS to be a basal ganglia disorder, but as research progressed, evidence suggested that PSS is related to the dysfunction of a network involving the basal ganglia, thalamus, cerebellum, and sensorimotor cortex (Tewari et al., 2017; Bologna and Berardelli, 2018; Brüggemann, 2021). The occurrence of spasticity is not only associated with damage to a specific brain structure, but may also be related to the reorganization of the distant cortex after brain injury and to abnormalities in the entire brain network. This was confirmed by our study, which found that the lesions causing PSS were functionally connected to brain regions far removed from the lesions and were even involved in a common brain network defined by positive connectivity to the bilateral putamen and globus pallidus. The results were highly sensitive and specific for PSS, and split-half replication demonstrated that the results were highly repeatability. This was consistent with other recent studies on lesion network mapping. In all cases, lesions causing the same symptoms were associated with particular brain regions. For example, lesions causing freezing of gait were connected to the cerebellum (Fasano et al., 2017), lesions causing cervical dystonia were associated with the cerebellum and somatosensory cortex (Corp et al., 2019), and lesions causing post-stroke depression were connected to the left dorsolateral prefrontal (Padmanabhan et al., 2019). The present study found that the putamen and globus pallidus had the most sensitive and specific association with lesions leading to PSS.

The putamen and globus pallidus are parts of the basal ganglia, separated from the caudatum and thalamus by the internal capsule. MRI-based localization of spasticity-associated brain regions showed a greater density of lesions in the putamen, internal capsule, insula, and thalamus. When comparing the degree of lesion overlap in stroke patients with spasticity, the putamen was found to delineate the presence or absence of spasticity, suggesting that the putamen was the “spasticity” region and not simply the common location of stroke injury (Bradnam et al., 2012). In the cortex-basal ganglia pathway, the putamen and caudatum form the neostriatum, which receives information from the frontal lobe and sensorimotor cortex, and then sends signals of motor initiation through the globus pallidus (Li et al., 2020). When a patient with PSS initiates movement, the patient experiences impairments in movement timing, speed, amplitude, frequency, preparation, and instruction. However, the neostriatum has insufficient ability to integrate this abnormal motor pattern and sensory input. Therefore, it is compensated by overactivation, which further affects the excitability of the cortico-neostriatal-pallidum-thalamo-cortical loop. This ultimately leads to abnormal projections from the cortex to the spinal cord (Bar-Gad et al., 2003; Fogelson et al., 2006). This study, we found a positive correlation between the functional connectivity of lesions with the putamen and globus pallidus in patients with PSS and the degree of spasticity, indicating that the more severe the spasticity, the stronger the functional connectivity of lesions with the putamen and globus pallidus in patients. It indicates that there was compensation by increased contact with these areas, which was consistent with the above findings, that is, the abnormal function of the cortex-basal ganglia-thalamus-cortex motor loop was closely related to the pathogenesis of PSS. The putamen and globus pallidus play central anatomical and functional roles in this loop.

In addition, a recent study found that lesions, transcranial magnetic stimulation targets and deep brain stimulation (DBS) targets in post-stroke depression converge in a common brain network, suggesting that lesions and treatment targets were in the same brain network and that invasive and non-invasive brain stimulation targeted the same network to treat the same symptoms (Joutsa et al., 2018b; Siddiqi et al., 2021). Confirmed lesion network mapping results show promise in identifying new, symptom-specific therapeutic targets, and can be used as a measure of effectiveness in identifying therapeutic targets. Our lesion network mapping results showed that the putamen and globus pallidus were the most sensitive and specific brain regions for PSS, suggesting that the putamen and globus pallidus are potential therapeutic targets for PSS. To the best of our knowledge, the optimal brain stimulation target for PSS is unknown. Globus pallidus interna DBS has shown some promise, but clinical studies have reported widely varying results, with some centers finding significant and sustained improvement in symptoms after DBS treatment, whereas others have found only slight or temporary improvement (Elias et al., 2018). In addition, globus pallidus interna DBS was not a specific therapeutic target for PSS and has been used to treat many other dystonia disorders. Our study found that the lateral part of the globus pallidus may be more correlated to PSS. Some studies have shown that the external globus pallidus was not only related to the input structures of the basal ganglia (striatum and subthalamic nucleus), but also to the main fiber innervation source of the output nucleus (globus pallidus internus and substantia nigra pars reticulata) (Kita, 2007). The external globus pallidus may be the center of the functional nuclei of the basal ganglia (Hegeman et al., 2016). Therefore, the globus pallidus implicated in our study may be expected to be a therapeutic target for PSS. Another promising stimulation target for PSS is the primary motor cortex (M1) (Málly and Dinya, 2008; Kuzu et al., 2021; Liu et al., 2021), which has become a major target for non-invasive brain stimulation in patients with PSS. Evidence suggests that the putamen is closely associated with the primary motor cortex, receiving all inputs from the primary sensorimotor cortex and most inputs from the premotor cortex (Cheung et al., 2016). Therefore, the putamen and M1 may belong to the same brain circuit, which affects putamen function by stimulating cortical M1 targets. This supports the concept that brain networks, rather than individual areas, may be the appropriate targets for brain stimulation. Future research should investigate whether our putamen, pallidum, and related connectivity networks are effective brain stimulation targets for improving PSS.

### Limitations

Previous studies have revealed some potential confounding factors in lesion network mapping, such as differences in age and sex between healthy subjects and patients and differences in fMRI processing methods (Boes et al., 2015; Horn et al., 2017), although these barely affect the results. However, there are still several limitations of this study that need to be addressed. First, lesion network mapping based on a group of healthy subjects cannot account for compensatory or adaptive reorganization changes in brain networks after brain injury (Desowska and Turner, 2019; Guggisberg et al., 2019). It seems possible to identify only the regions that may be functionally affected by the lesion, without considering compensatory network adaptations that may occur after brain injury. The functional neuroimaging abnormalities of the patients may represent the combination of functional changes directly caused by the lesions and secondary compensatory responses. Second, our study used a large-scale connectome dataset of healthy people (*n* = 1,000), which improves the reliability of connectivity estimation and provides a standardized template that can be used in neuroimaging research, but which does not take into account significant individual differences in functional connectivity. In other circumstances, functional connectivity in individual patients may also be important. Therefore, further studies need to collect the fMRI data from patients after stroke and combine this technique with resting-state functional connectivity data derived from these patients. This could be beneficial to understand the association between the structure and function of brain networks and to explore the reorganization mechanism of the brain network after stroke, as well as the individual differences among patients. Finally, although the patients in this study had limited lesion damage, the lesions were seldom localized in the gray matter. Disruption of white matter tract connectivity due to white matter damage may also contribute to PSS (Li and Francisco, 2015; Barlow, 2016; Plantin et al., 2019). Future studies should further analyze these lesions in conjunction with structural connectome datasets to explore regions with disruption of distal structural connectivity to the lesions and should further uncover the mechanisms underlying PSS occurrence. How best to combine the structural and functional connectomes of the brain to map the lesion network is a significant subject for future work. In addition, future multicenter studies with a large sample size of independent replication cohorts are needed to further examine the reproducibility of the lesion network of PSS.

## Conclusion

In conclusion, this study showed that heterogeneous lesions causing PSS are localized to a common brain network defined by connectivity to the bilateral putamen and globus pallidus. This network may involve the location of all lesions causing PSS. Our findings highlight that the putamen and globus pallidus may potentially be critical regions for PSS development. This complements previous neuroimaging studies on PSS and may help the early prevention of patients at high risk for PSS. However, further work is required to understand the pathophysiology of PSS-specific regions and to determine whether these regions can be used as new therapeutic targets for PSS.

## Data availability statement

The raw data supporting the conclusions of this article will be made available by the authors, without undue reservation.

## Ethics statement

The studies involving human participants were reviewed and approved by the 900th Hospital of Joint Logistic Support Force. The patients/participants provided their written informed consent to participate in this study.

## Author contributions

YQ, SQ, and XL conceived the study design and drafted the manuscript. XG and YT were responsible for data collection. SX and XW analyzed fMRI data and created diagrams. HL made a critical revision of the article. All authors participated in the final version of the manuscript.

## Footnotes

1
https://doi.org/10.7910/DVN/25833

2
https://www.fil.ion.ucl.ac.uk/spm/

3
https://www.nitrc.org/projects/mricro

4
https://opensourcelibs.com/lib/spm12
